# Transposon sequencing reveals the essential gene set and genes enabling gut symbiosis in the insect symbiont *Caballeronia insecticola*

**DOI:** 10.1093/ismeco/ycad001

**Published:** 2024-01-10

**Authors:** Romain Jouan, Gaëlle Lextrait, Joy Lachat, Aya Yokota, Raynald Cossard, Delphine Naquin, Tatiana Timchenko, Yoshitomo Kikuchi, Tsubasa Ohbayashi, Peter Mergaert

**Affiliations:** Université Paris-Saclay, CEA, CNRS, Institute for Integrative Biology of the Cell (I2BC), Gif-sur-Yvette 91198, France; Université Paris-Saclay, CEA, CNRS, Institute for Integrative Biology of the Cell (I2BC), Gif-sur-Yvette 91198, France; Université Paris-Saclay, CEA, CNRS, Institute for Integrative Biology of the Cell (I2BC), Gif-sur-Yvette 91198, France; Université Paris-Saclay, CEA, CNRS, Institute for Integrative Biology of the Cell (I2BC), Gif-sur-Yvette 91198, France; Université Paris-Saclay, CEA, CNRS, Institute for Integrative Biology of the Cell (I2BC), Gif-sur-Yvette 91198, France; Université Paris-Saclay, CEA, CNRS, Institute for Integrative Biology of the Cell (I2BC), Gif-sur-Yvette 91198, France; Université Paris-Saclay, CEA, CNRS, Institute for Integrative Biology of the Cell (I2BC), Gif-sur-Yvette 91198, France; Bioproduction Research Institute, National Institute of Advanced Industrial Science and Technology (AIST), Hokkaido Center, Sapporo 062-8517, Japan; Université Paris-Saclay, CEA, CNRS, Institute for Integrative Biology of the Cell (I2BC), Gif-sur-Yvette 91198, France; Institute for Agro-Environmental Sciences, National Agriculture and Food Research Organization (NARO), Tsukuba 305-8604, Japan; Université Paris-Saclay, CEA, CNRS, Institute for Integrative Biology of the Cell (I2BC), Gif-sur-Yvette 91198, France

**Keywords:** gut symbiosis, Caballeronia insecticola, Riptortus pedestris, Tn-seq, essential gene, fitness gene, Burkholderia sensu lato, genome comparison

## Abstract

*Caballeronia insecticola* is a bacterium belonging to the *Burkholderia* genus *sensu lato*, which is able to colonize multiple environments like soils and the gut of the bean bug *Riptortus pedestris*. We constructed a saturated *Himar1* mariner transposon library and revealed by transposon-sequencing that 498 protein-coding genes constitute the essential genome of *Caballeronia insecticola* for growth in free-living conditions. By comparing essential gene sets of *Caballeronia insecticola* and seven related *Burkholderia s.l.* strains, only 120 common genes were identified, indicating that a large part of the essential genome is strain-specific. In order to reproduce specific nutritional conditions that are present in the gut of *Riptortus pedestris*, we grew the mutant library in minimal media supplemented with candidate gut nutrients and identified several condition-dependent fitness-defect genes by transposon-sequencing. To validate the robustness of the approach, insertion mutants in six fitness genes were constructed and their growth deficiency in media supplemented with the corresponding nutrient was confirmed. The mutants were further tested for their efficiency in *Riptortus pedestris* gut colonization, confirming that gluconeogenic carbon sources, taurine and inositol, are nutrients consumed by the symbiont in the gut. Thus, our study provides insights about specific contributions provided by the insect host to the bacterial symbiont.

## Introduction

Essential genes in a bacterial genome are genes that are indispensable to support cellular life. Together, they constitute a minimal gene set required for a living bacterium. Essential genes are a subset of fitness genes that contribute to the reproduction of an organism independently of its environment. Thus, a fitness gene can be defined as any gene whose perturbation causes a proliferation defect and an essential gene is the extreme case when there is no proliferation at all [1, 2]. In contrast to the environment-independent essential and fitness genes, conditionally essential or fitness genes are only required for proliferation under specific conditions. Identification of (conditionally) essential and fitness genes of a bacterium contributes to the understanding of the molecular factors enabling its lifestyle and, from an applied point of view, it can help in designing new ligands for disease management or development of improved bio-stimulants [3, 4].

Multiple methods have been developed to identify essential and fitness genes at the whole-genome scale. Among them is the systematic deletion of all possible genes and their one-by-one precise phenotyping [5, 6]. This method is laborious, making it difficult to undertake for most bacterial species. However, the recent development of Transposon-sequencing (Tn-seq) and related methods has provided an efficient and fast solution to determine the essential genome that is accessible for a large number of bacterial species. Tn-seq allows to perform high-throughput genetic screens and to quantify in a single selection experiment the fitness impact of all genes of a genome in a condition of interest. Tn-seq uses saturated transposon-mutant populations, determines the relative abundance of all mutants in the population by high-throughput sequencing, and compares these abundances in different growth conditions [7]. The analysis of the genome-wide mutation frequency in the population grown in standard conditions identifies the essential and fitness genes, whereas the determination of altered mutation frequencies between conditions identifies the conditionally essential genes. By quantifying gene fitness by Tn-seq in many different specific environments, a fitness landscape of a bacterium in a large diversity of conditions can be created [8-10].

*Burkholderia sensu lato* (*s.l.*), corresponding to the former *Burkholderia* genus and belonging to the *Betaproteobacteria*, is a large group of pathogenic, phytopathogenic, symbiotic, and environmental bacteria composed of eight different genera: *Paraburkholderia*, *Robbsia*, *Pararobbsia*, *Mycetohabitans*, *Caballeronia*, *Trinickia*, *Burkholderia sensu stricto*, and *Burkholderia cepacia* complex [11]. *Caballeronia insecticola* (formerly *Burkholderia insecticola*) is able to colonize multiple environments like soil or the gut of the bean bug *R. pedestris* and related stinkbug species [12-14]. In this latter environment, *C. insecticola* is involved in a mutualistic relationship with the insects. The symbiotic bacteria colonize an exclusive niche composed of crypts located in the posterior midgut region of the insect where, starting from a limited number of infecting bacteria acquired from the environment through feeding, a massive extracellular population is established in the midgut crypts in a few days [15, 16]. In return, *C. insecticola* enhances the insect’s development, reproduction, and immunity [17, 18]. A transcriptome analysis of *C. insecticola* in free-living growth and during colonization of the *R. pedestris* gut crypts suggested that *C. insecticola* is fed in the gut with specific nutrients and also recycles host metabolic wastes, and in return, the bacterial symbiont provides the host with essential nutrients limited in the insect food, contributing to the rapid growth and enhanced reproduction and immunity of the bean bug host [13].

Here, a saturated *Himar1* mariner transposon mutant library of *C. insecticola* allowed us to identify the essential gene set for *in vitro* growth in standard rich medium and in a defined minimal medium (MM). In addition, to mimic specific nutrient conditions that are likely present in the insect symbiotic organ, this mutant library was grown in minimal medium supplemented with specific gut nutrients and we identified several condition-dependent fitness genes. Thus identified candidate genes for growth on different carbon sources were selected for mutagenesis and the constructed mutants were tested for growth on the relevant nutrient sources and for their capacity to colonize the *R. pedestris* symbiotic gut organ in mono inoculation or in co-inoculation with the wild-type (WT) bacteria.

## Materials and methods

### Bacterial strains and growth conditions

*Caballeronia insecticola* strains were cultured at 28°C in Yeast-Glucose medium (YG: 5 g/l yeast extract; 1 g/l NaCl; 4 g/l glucose) for routine use or in MM supplemented with various carbon, sulfur, or nitrogen sources for the Tn-seq screens (Supplementary Data Set S1). *Caballeronia insecticola* RPE75 is a spontaneous rifampicin (Rif)-resistant derivative of the WT strain RPE64 [12]. For standard molecular microbiology purposes, *Escherichia coli* strains DH5α, HB101, WM3064, MFDpir, S17-1λpir, and their derivatives were grown in LB medium (5 g/l yeast extract, 10 g/l tryptone, 5 g/l NaCl) at 37°C. Growth of the MFDpir and WM3064 strains that are ∆*dapA* derivatives, auxotroph for diaminopimelic acid (DAP) synthesis, required the supplement of 300 μg/ml DAP to the medium. When appropriate, antibiotics were added to the medium in the following concentrations: 50 μg/ml kanamycin (Km) for *E. coli* and 30 μg/ml for *C. insecticola*; 25 μg/ml chloramphenicol; 30 μg/ml Rif; 100 μg/ml ampicillin. For solid agar plates, the media were supplemented with 1.5% agar.

### Generation of a *Caballeronia insecticola* RPE75 *Himar1* transposon library

A *Himar1* transposon library was generated following the methods described before [19]. Detailed procedures for the construction and quality control of the library are provided in the Supplementary Text.

### Tn-seq screening of the *Himar1* transposon library for growth with different nutrients

An aliquot of the Tn-seq library was 100-fold diluted to reach a suspension of 2 × 10^8^ cfu/ml. Hundred microliter of this dilution was inoculated into 20 ml of a growth medium, supplemented with Rif and Km, to obtain an initial inoculum of 10^6^ cfu/ml (OD_600nm_ ≈ 0.0015). Eleven growth conditions were tested: YG medium and 10 different MM supplemented with various carbon, sulfur, or nitrogen sources. The assembly from stock solutions and the composition of the MM medium are provided in Supplementary Data Set S1. Cultures were incubated at 28°C, with shaking at 180 rpm. When the cultures reached an OD_600nm_ ≈ 1, corresponding to ~9–10 generations of multiplication, bacteria were collected by centrifugation at 4000 rpm for 20 min at 4°C and the pellets were stored at −20°C until DNA extraction. Each condition was performed in triplicates.

### DNA extraction and preparation of the high-throughput sequencing libraries

Genomic DNA was extracted from the bacterial pellets using the MasterPure™ Complete DNA and RNA purification kit (Epicentre) according to the manufacturer’s instructions. Preparation, concentration determination, and quality control of the high-throughput sequencing libraries were done following procedures described before [19] and detailed in the Supplementary Text.

### Sequencing and sequence data treatment

Sequencing and sequence data treatment were done as described [19], using the reference genome of *C. insecticola* (accession n° NC_021287.1 (Chromosome 1), NC_021294.1 (Chromosome 2), NC_021288.1 (Chromosome 3), NC_021289.1 (Plasmid 1), NC_021295.1 (Plasmid 2)). Detailed information is provided in the Supplementary Text.

### Identification of (conditionally) essential genes by Transit software

Tn-seq sequencing data were handled by TRANSIT Version 3.2.0 [20] using Hidden Markov Model (HMM) analysis to determine essentiality within a single condition and resampling analysis to compare two conditions. Detailed information is provided in the Supplementary Text

### Homology-based comparison between *Burkholderia sensu lato* species

OrthoVenn2 [21] was used to compare the essential genomes of eight selected *Burkholderia* species. We used *E*-value of 1e−15 and inflation value of 1.5 as analysis parameters. Data from the following studies were used to establish the lists of the essential genome for each of seven other *Burkholderia* species besides *C. insecticola*: *Burkholderia pseudomallei* strain K96243 [22], *Burkholderia cenocepacia* strain J2315 [23], *B. cenocepacia* strain H111 [24], *B. cenocepacia* strain K56-2 [25], *Burkholderia thailandensis* strain E264 [26], *Burkholderia vietnamiensis* strain LMG10929 [27], and *Paraburkholderia kururiensis* strain M130 [27].

### Construction of *Caballeronia insecticola* RPE75 insertion mutants and fluorescent protein tagged strains

For insertion mutagenesis of *C. insecticola* RPE75, internal fragments (300–600 bp) of the target gene were amplified by PCR (Supplementary Table S1) and cloned into the pVO155-p*nptII*-*GFP* vector using restriction enzymes *Sal*I-*Xba*I or *Xho*I-*Xba*I and ligation or alternatively by Gibson assembly cloning. Constructs were introduced in *E. coli* DH5α by heat shock transformation and selection with Km. Candidate colonies were confirmed by colony-PCR and Sanger sequencing (Eurofins Genomics). The plasmid construct was transferred to the recipient *C. insecticola* RPE75 strain by triparental conjugation, with the *E. coli* DH5α donor strain and the *E. coli* HB101.pRK600 helper strain. Transconjugants were selected with Rif and Km. Candidate *C. insecticola* mutants were verified by colony-PCR and by checking the GFP fluorescence. A mScarlett-I-tagged strain of *C. insecticola* was created by introducing a Tn7-Scarlet transposon using triparental mating as described [28, 29] (Supplementary Text).

**Table 1:** Relevant features of the genome of *Caballeronia insecticola*.

	Size (bp)	N° of TA sites	N° of genes	N° of TA sites in genes	N° of genes without TA	Gene size without TA (bp)
Chromosome 1	3 013 410	45 986	2803	27 310	34	25–199
Chromosome 2	1 465 356	23 340	1337	14 103	18	53–170
Chromosome 3	900 830	14 869	790	9368	3	106–164
Plasmid 1	1 275 199	20 575	1157	13 056	8	61–143
Plasmid 2	309 692	5965	262	3439	0	—
Whole genome	6 964 487	110 735	6349	67 276	63	25–199

### Bacterial growth determination on different nutrients

To determine the growth capacity of the *C. insecticola* mutants on different carbon sources, precultures of tested strains were grown in MM medium. Overnight grown cultures were diluted to an OD_600nm_ = 0.3 in fresh medium and grown until they reached OD_600nm_ ≈ 1. The cells were pelleted by centrifugation, resuspended to an OD_600nm_ = 0.05 in fresh medium with the tested carbon source. These cell suspensions were dispatched in a 96-well plates, which were incubated in a SPECTROstar Nano plate incubator (BMG Labtech). The growth of the cultures in the wells was monitored by measuring the OD_600nm_ and data points were collected every hour for 48 h. Plates were incubated at 28°C with double orbital shaking at 200 rpm. Data and growth curves were analyzed using Microsoft Excel.

### Insect rearing and inoculation tests

Insect rearing and inoculation tests were conducted as before [30]. At 3 and 5 days postinoculation, insects, at the stage of the end of the second instar nymphs or the third instar, respectively, were dissected. The colonization rate of mono-inoculated insects was estimated by fluorescent signal detection of colonizing bacteria with GFP or mScarlett-labeled fluorescent proteins. For co-inoculation experiments, M4 region samples were homogenized in PBS solution and bacteria in suspension were counted by flow cytometry, using the fluorescent tags to determine the relative abundance of the two inoculated strains (see Supplementary Text for details).

## Results

### Construction and sequencing of a Tn-seq library of *Caballeronia insecticola*

We mutagenized *C. insecticola* with a *Himar1* mariner transposon that selectively inserts in TA sites of the genome. The five replicons of the *C. insecticola* genome, Chromosomes 1–3 and Plasmids 1 and 2, present a total DNA content of 6.96 Mbp and contain 6349 annotated genes [31]. Among the 110 735 TA sites present in the genome, 67 276 of them are located in the annotated genes. Among the 6349 annotated genes, 6286 possess TA sites; hence, *Himar1*-based mutagenesis covers ~99.01% of genes. The genes lacking TA sites (63 genes) are short to very short open reading frames varying in length from 25 to 199 nucleotides and encoding mostly peptides of unknown function (Table 1). On the other hand, genes with TA sites have a mean of 10.6 TA sites in their sequence. Thus, as the proportion of genes without TA sites in the genome is small (0.99%) and the large majority of genes have a high number of TA sites, it was feasible to produce a genome-wide *C. insecticola* transposon insertion library with a good coverage using the *Himar1* mariner transposon. A large-scale mutagenesis resulted in ~2.5 × 10^8^ independent clones, representing an over 2000-fold coverage of all theoretically possible mutants, with 80% of the TA sites located within a gene carrying insertions.

**Figure 1:**
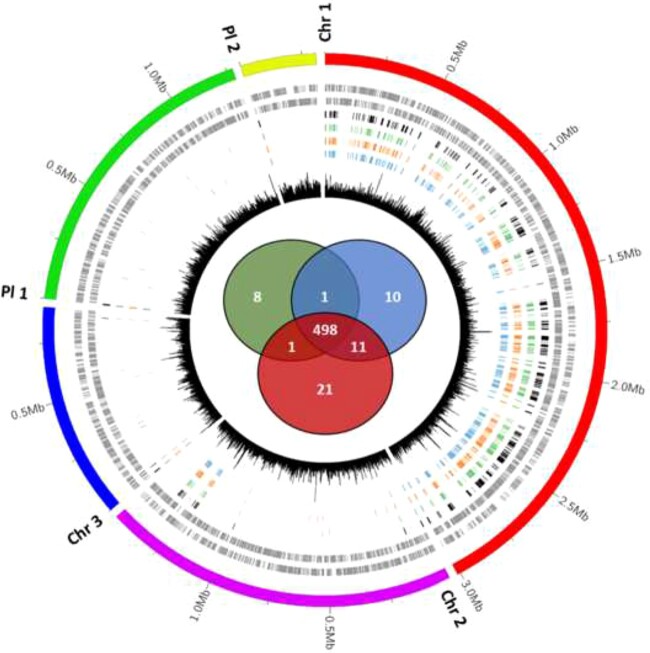
*Caballeronia insecticola* essential genes; circular representation of the genome of *C. insecticola*, the markings outside the outer circle represent genome positions (in Mb) for each chromosome or plasmid; Chr 1 is Chromosome 1, Chr 2 is Chromosome 2, Chr 3 is Chromosome 3, Pl 1 is Plasmid 1, and Pl 2 is Plasmid 2; the second and third tracks represent CDS on the forward and reverse strand, respectively; subsequent tracks 4, 5, 6, and 7 represent, respectively, the essential genes that are common to the YG, MM glucose, and MM succinate conditions, the essential genes specific for YG, MM succinate, and MM glucose media; the innermost track 8 shows the number of TA sites per 1000 bp; the Venn diagram in the center represents the number of essential genes identified for each medium: top left, YG; top right, MM glucose; bottom, MM succinate.

The mutant library was grown in three different standard growth media: a rich medium (YG) and a defined MM with glucose or succinate as carbon source, and cultures were subjected to Tn-seq. The essential genome was determined using the HMM in Transit software [20] and considering only the genes that are common to the three conditions as part of the essential gene set. This analysis classified the majority of the genes (92%) as nonessential and 498 (8%) protein-coding genes as essential (Fig. 1; Supplementary Data Set S2). This proportion of the *C. insecticola* genome identified as its essential genome is very similar to what is found in other bacteria [32]. These 498 genes were mostly located on Chromosome 1 (464 genes or 93% of total). Another 22 genes (4.5%) were on Chromosome 2 and only 5, 4, and 3 genes were found on Chromosome 3, Plasmid 1, and Plasmid 2, respectively.

**Figure 2:**
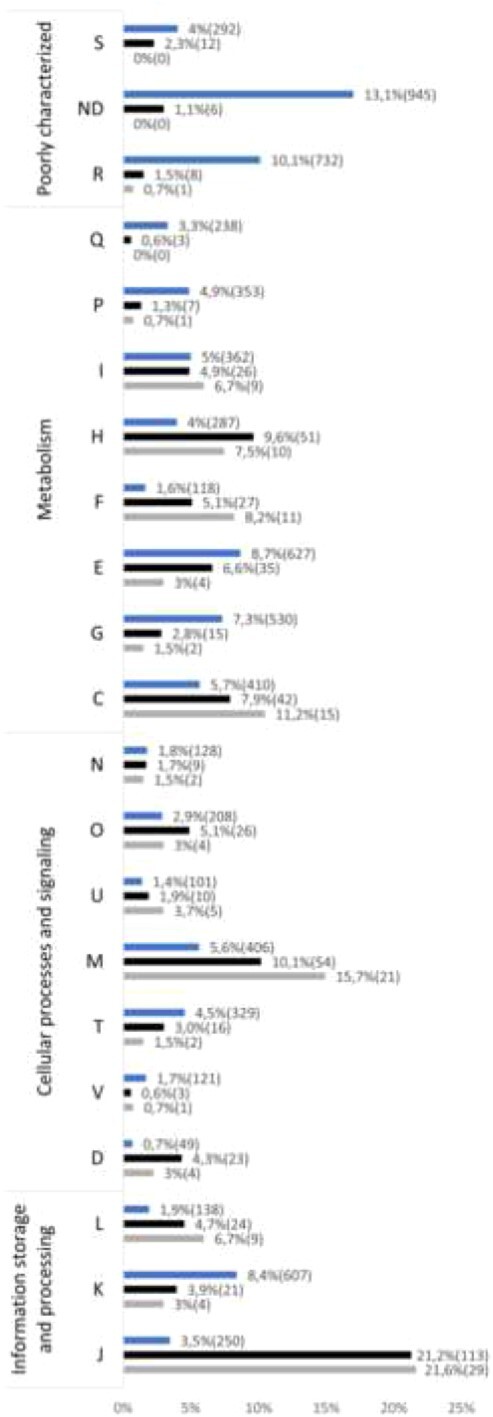
Distribution of *C. insecticola* essential genes in COG categories; the categories are S, function unknown; ND, not determined; R, general function prediction only; Q, secondary metabolites biosynthesis—transport—catabolism; P, inorganic ion transport—metabolism; I, lipid transport—metabolism; H, coenzyme transport—metabolism; F, nucleotide transport—metabolism; E, amino acid transport—metabolism; G, carbohydrate transport—metabolism; C, energy production—conversion; N, cell motility; O, posttranslational modification—protein turnover—chaperones; U, intracellular trafficking—secretion—vesicular transport; M, cell wall—membrane—envelope biogenesis; T, signal transduction mechanisms; V, defense mechanisms; D, cell cycle control—cell division—chromosome partitioning; L, replication—recombination—repair; K, transcription; J, translation—ribosomal structure—biogenesis; for each category, the number of genes and the percentage that they represent are indicated; top histograms indicate the whole genome of *C. insecticola*, center histograms indicate the essential genome of *C. insecticola*, and lower histograms indicate the common essential genes to eight *Burkholderia s.l.* species.

**Figure 3:**
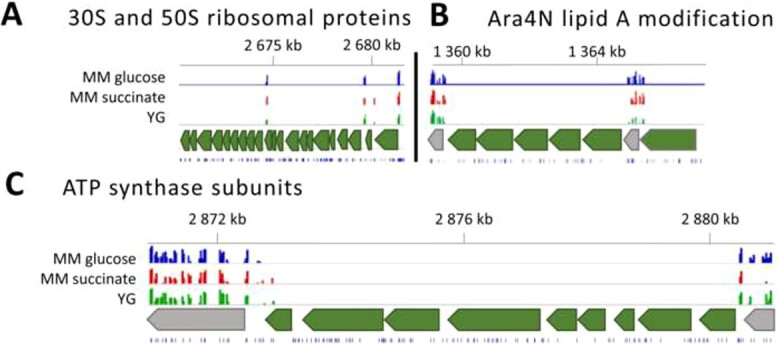
IGV plots for genomic regions containing selected *C. insecticola* essential genes; (A) 30S and 50S ribosomal proteins encoding region; (B) Ara4N lipid A modification gene cluster; (C) ATP synthase subunits encoding region; tracks, from bottom to top: position of TA sites, region of interest and its flanking neighbors, histogram of insertion counts at TA sites for the indicated experimental conditions, genome positions (in kb) on Chromosome 1.

According to clusters of orthologous genes (COG) classification [33], the most represented category of essential genes was related to translation, ribosomal structure, and biogenesis (J category) (Fig. 2). Genes encoding for 30S and 50S ribosomal proteins are examples of this functional class (Fig. 3). The cell wall biogenesis category (M category), the coenzyme transport and metabolism category (H category), and the energy conversion and production category (C category) also contain many essential functions. Examples in these three categories are genes involved in lipid A and peptidoglycan biosynthesis, the heme biosynthesis pathway, or genes involved in respiration, like the ATP synthase subunits (Fig. 3) and the Respiratory Complex I subunits. Other highly represented categories are amino acid metabolism (E category) with the genes taking part in the L-histidine biosynthesis as examples or the transcription machinery (K category) with the RNA polymerase subunits. The distribution of the essential genes among the COG categories is very different compared with all the annotated genes in the genome (Fig. 2), demonstrating that our analysis identified specific functions as essential.

Of note, in the cell cycle control, cell division, and chromosome partitioning category (D category) are the genes involved in the replication and partitioning of the genome. Chromosome 1 has a typical organization similar to the principal chromosome of *B. cenocepacia*, carrying on the replication origin locus the genes *rpmH* (BRPE64_RS14035), *rnpA* (BRPE64_RS14030), *dnaA* (BRPE64_RS00005), *dnaN* (BRPE64_RS00010), and *gyrB* (BRPE64_RS00015), and on a nearby locus the chromosome partitioning genes *parA* (BRPE64_RS13400) and *parB* (BRPE64_RS13395) [34, 35]. Except for *rpmH* that has no TA sites, all these genes were found to be essential in agreement with their crucial role in the replication and thus persistence of Chromosome 1. On the other hand, Chromosomes 2 and 3 have, similarly to Plasmids 1 and 2, a plasmid-like replication origin locus, with its own distinct *parABS* system and a plasmid-like replication protein (Chromosome 2: BRPE64_RS14050, BRPE64_RS14055, BRPE64_RS14060; Chromosome 3: BRPE64_RS20740, BRPE64_RS20745, BRPE64_RS20750; Plasmid 1: BRPE64_RS24690, BRPE64_RS24695, BRPE64_RS24700; Plasmid 2: BRPE64_RS30485, BRPE64_RS30490, BRPE64_RS30495). Thus, Chromosomes 2 and 3 have plasmid-like features although they carry several essential genes. According to a new classification of bacterial replicons, Chromosomes 2 and 3 should be considered as “chromids” [36-38]. Interestingly, each of these *parABS* and replication protein-encoding genes were found to carry no transposon mutations, indicating that these replicons require their own cognate machinery for replication and partition. While the three chromosomes carry other essential genes, only the genes implicated in replication and partitioning are free of mutations in the two plasmids. Thus in this case, the absence of transposon insertions in these genes does not mean that they are essential for cell viability but rather essential for the maintenance of the replicon. On the contrary, since the chromosomes contain other essential genes, these replicons and their replication/partitioning functions are essential for viability.

Taken together, the pathways of the *C. insecticola* essential genome highlight cellular functions, like transcription, translation, energy production, cell envelope biosynthesis, and cell cycle, known to represent vital functions for bacteria [39, 40].

### Comparative analyses of essential genes in eight *Burkholderia s.l.* species

Tn-seq techniques were used to identify essential genes in pathogenic *Burkholderia* species including *B. pseudomallei* strain K96243 [22], *B. cenocepacia* strain J2315 [23], *B. cenocepacia* strain H111 [24], and *B. cenocepacia* strain K56-2 [25]; in the plant-associated species *B. vietnamiensis* strain LMG10929 and *Paraburkholderia kururiensis* strain M130 [27]; as well as in the environmental species *B. thailandensis* strain E264 [26]. These studies revealed 505, 383, 339, 493, 620, 700, and 406 essential protein-coding genes in these bacteria, respectively. The comparison of these gene sets with the *C. insecticola* essential genes allowed us to identify a total of 120 essential genes shared between all eight species (Supplementary Fig. S1; Supplementary Data Set S3). If essential genes shared by seven out of the eight species are considered, the number of common genes increases to 231 (Supplementary Fig. S1) and the number of pairwise shared essential genes ranges from 195 to 412 genes with as a mean 291 genes (Supplementary Fig. S2). Among the 37 COG that are essential in at least four of the considered species but not in *C. insecticola*, we found for five clusters that the *C. insecticola* genome lacked the corresponding gene. In 26 cases, the gene was present but nonessential, and in six cases, the gene was duplicated in the genome.

Considering the 120 genes that are commonly essential to all eight species, an important part of them ensures functions related to the translation process (J category), the cell wall biosynthesis (M category), the coenzyme transport and metabolism (H category), or the energy production (C category) (Fig. 2). Examples of essential genes common to these eight bacterial species encode ribosomal subunits, initiation factors of translation, cell division and chromosome replication, ATP synthase and respiratory chain subunits, peptidoglycan biosynthesis enzymes or enzymes involved in lipid A biosynthesis. Remarkably, among the latter are part of the genes of the *arnBCA_1_A_2_DarnT* cluster (BRPE64_RS06345–BRPE64_RS06375 in *C. insecticola*), encoding the biosynthesis of 4-amino-4-deoxy-L arabinose (Ara4N) and its transfer to the lipid A moiety of lipopolysaccharide (LPS) (Fig. 3B). This gene cluster was in an independent approach found to be essential for viability in *B. cenocepacia* strain K56-2 [41]. The Ara4N modification of LPS mediates resistance against cationic antimicrobial peptides (AMPs) in many gram-negative bacteria, including *Burkholderia* species [42]. In the γ-proteobacteria *Salmonella* and *Pseudomonas*, the Ara4N modification is not essential and not constitutively present in lipid A but is introduced upon sensing of AMPs in the environment [43-46]. In contrast, the essentiality of the Ara4N modification in *Burkholderia s.l.* spp. suggests that the Ara4N lipid A modification is constitutive. Indeed, the lipid A structure of *B. cenocepacia* and *C. insecticola* is modified with one or two Ara4N moieties [41, 46]. As demonstrated for *B. cenocepacia*, the Ara4N modification is required for LPS export by the Lpt transporter that transfers fully assembled LPS from the inner membrane to the outer membrane and that has an absolute specificity for Ara4N carrying LPS molecules [47].

### Conditional fitness defect genes

In a previous study, the comparison of the transcriptomes of the free-living and *R. pedestris* midgut-colonizing *C. insecticola* highlighted up or downregulated metabolic pathways [13]. Transporters or metabolic pathways of diverse sugars such as rhamnose and ribose, and sulfur compounds like sulfate and taurine (C_2_H_7_NO_3_S) were upregulated in the midgut-colonizing bacteria. Moreover, glycolytic pathways were downregulated and the gluconeogenesis pathway was upregulated. These data indicate that symbiotic bacteria could depend on particular sources provided by the insect. We used Tn-seq to identify key genes in *C. insecticola* for growth on these nutrients.

**Figure 4:**
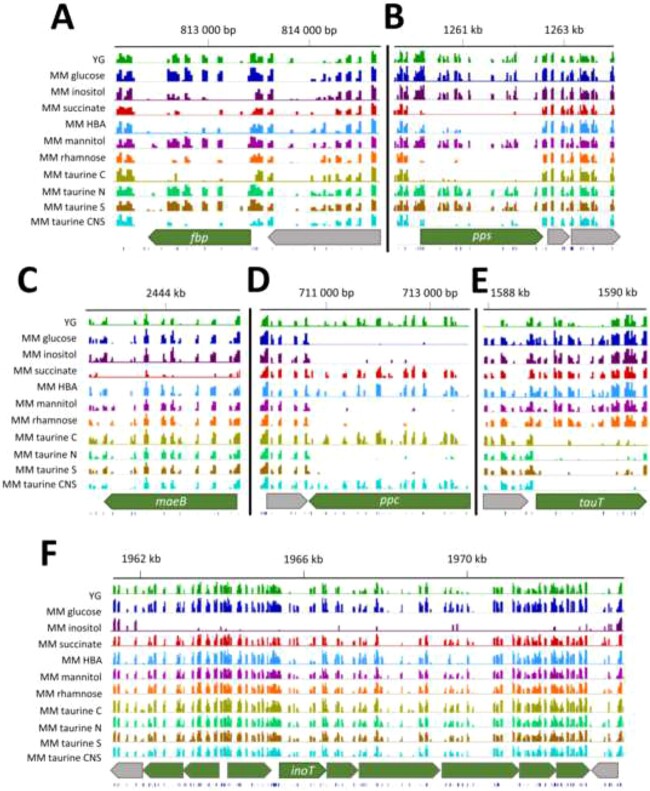
IGV plots of genomic regions carrying condition-specific fitness genes; (A) fructose-bisphosphatase encoding gene (*fbp*); (B) phosphoenolpyruvate synthase encoding gene (*pps*); (C) malic enzyme encoding gene (*maeB*); (D) phosphoenolpyruvate carboxylase encoding gene (*ppc*); (E) chloride channel protein and potential taurine transporter encoding gene (*tauT*); (F) myo-inositol utilization genes including the ABC transporter permease (*inoT*); tracks, from bottom to top: position of TA sites; gene organization in the region of interest with fitness genes and their flanking neighbors; histograms of insertion counts at TA sites for the indicated experimental conditions; genome positions (in kb) on Chromosome 1; taurine C, taurine as carbon source; taurine N, taurine as nitrogen source; taurine S, taurine as sulfur source; taurine CNS, taurine as carbon, nitrogen, and sulfur source.

To mimic the specific nutrient conditions that can be found in the insect symbiotic organ, the *C. insecticola* transposon mutant library was grown in MM supplemented with different compounds. Glucose, 3-hydroxybutyric acid (HBA), mannitol, succinate, myo-inositol, or rhamnose were used as the only carbon sources. Taurine was used as sole carbon source, or as sole nitrogen or sulfur source, or as sole carbon/nitrogen/sulfur source. Tn-seq data were obtained for each growth condition, and we used the resampling method analysis from the Transit software package to identify the fitness defect genes for each condition, using MM with glucose or YG rich medium as the reference control condition (Supplementary Data Set S4). By these comparisons, we identified sets of condition-dependent fitness defect genes for each growth conditions (Fig. 4 and Supplementary Fig. S3; Supplementary Data Set S4). Examples are amino acid biosynthesis genes that are not fitness defect genes in rich medium, but are fitness defect genes in MM lacking any source of amino acids. Genes encoding glycolytic enzymes, like the phosphoenolpyruvate carboxylase *ppc* (BRPE64_RS03260), are fitness defect genes in MM supplemented with glycolytic substrates glucose, rhamnose, mannitol, or myo-inositol but not with gluconeogenic substrates succinate, HBA, and taurine. Inversely, the gluconeogenesis-specific genes *pps* (BRPE64_RS05810), encoding phosphoenolpyruvate synthase, and *fbp* (BRPE64_RS03750), encoding fructose-1,6-bisphosphatase, are fitness defect genes in MM with gluconeogenic substrates but not in MM with glycolytic substrates. *maeB* (BRPE64_RS11265), encoding the malic enzyme, is a fitness defect gene only in MM with succinate. Some genes appear as fitness defect in very specific conditions only. Examples are a contiguous cluster of nine genes encoding an ATP-binding cassette (ABC) transporter and involved in myo-inositol assimilation into the tricarboxylic acid cycle (TCA) cycle and gluconeogenesis/glycolysis (BRPE64_RS09045–BRPE64_RS09085) that has fitness defect only in the growth on myo-inositol condition. The gene BRPE64_RS07345, incompletely annotated as encoding a chloride channel, shows a fitness defect specifically in MM supplemented with taurine as carbon source, as nitrogen source, as sulfur source, or as carbon/nitrogen/sulfur source. This suggests that the BRPE64_RS07345-encoded chloride channel protein is a taurine uptake transporter, which we named TauT. TauT is unlike known bacterial taurine transporters, which are ABC transporters [48] or Tripartite ATP-independent periplasmic transporters [49]. Interestingly, the mammalian taurine transporter is a chloride-dependent channel. Gene cluster BRPE64_RS16735–BRPE64_RS16785 (11 genes) is specifically required for growth on rhamnose and codes for an ABC uptake transporter and the enzymes that incorporate rhamnose into pyruvate metabolism. The genes BRPE64_RS05370 and BRPE64_RS05375 encoding the subunits of 3-oxoacid CoA transferase allow to assimilate HBA into the TCA cycle and are specifically required for growth on HBA. Finally, genes BRPE64_RS02530–BRPE64_RS02555 are specifically required for growth on mannitol and codes for an ABC uptake transporter.

### Confirmation of Tn-seq results by mutagenesis of selected conditional fitness-defect genes

To confirm the Tn-seq results, insertion mutants of *C. insecticola* were constructed in a set of six selected fitness genes (Fig. 4) for specific nutrient conditions, and their ability to grow on the corresponding nutrient was evaluated. These chosen genes were the above-mentioned gluconeogenesis genes *fbp*, *pps*, and *maeB*, the glycolysis-specific gene *ppc*, the newly discovered putative taurine transporter gene *tauT*, and the myo-inositol transporter gene *inoT*.

**Figure 5:**
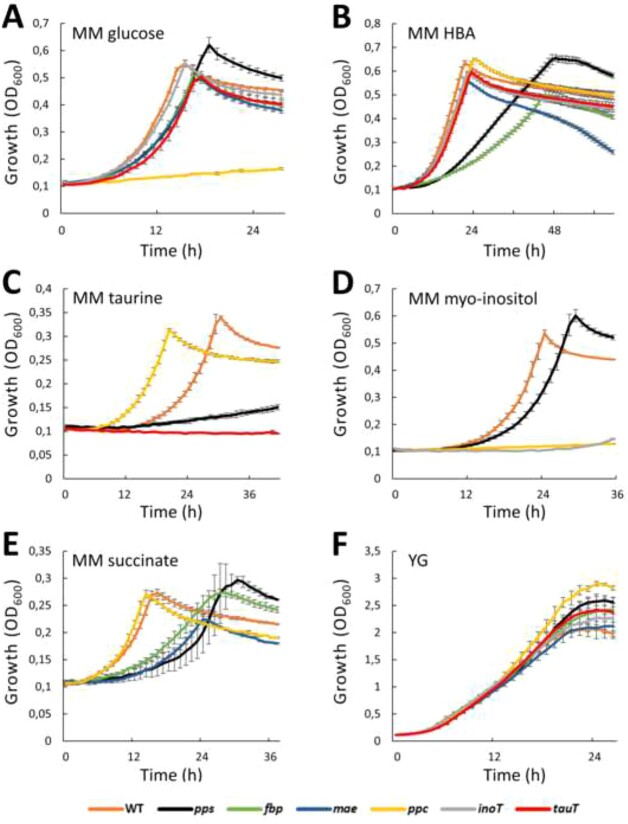
Growth curves of *C. insecticola* WT and metabolic mutants in different media; (A) MM with glucose; (B) MM with HBA; (C) MM with taurine as carbon source; (D) MM with myo-inositol; (E) MM with succinate; (F) YG medium. *X*-axis, time of growth in hours; *Y*-axis, growth measured as OD_600_; error bars are standard deviation.

Growth of the mutants in MM with the relevant carbon sources and in the rich medium YG as a control allowed us to confirm the above Tn-seq results (Fig. 5). As expected and indicated by Tn-seq, all mutants grew well in YG. The growth of the *fbp* and *pps* mutants was affected in MM with succinate, HBA, and taurine as carbon source, the *maeB* mutant in MM with succinate, the *ppc* mutant in MM with glucose and myo-inositol, the *tauT* mutant in MM with taurine as carbon source, and the *inoT* mutant in MM with myo-inositol.

**Figure 6:**
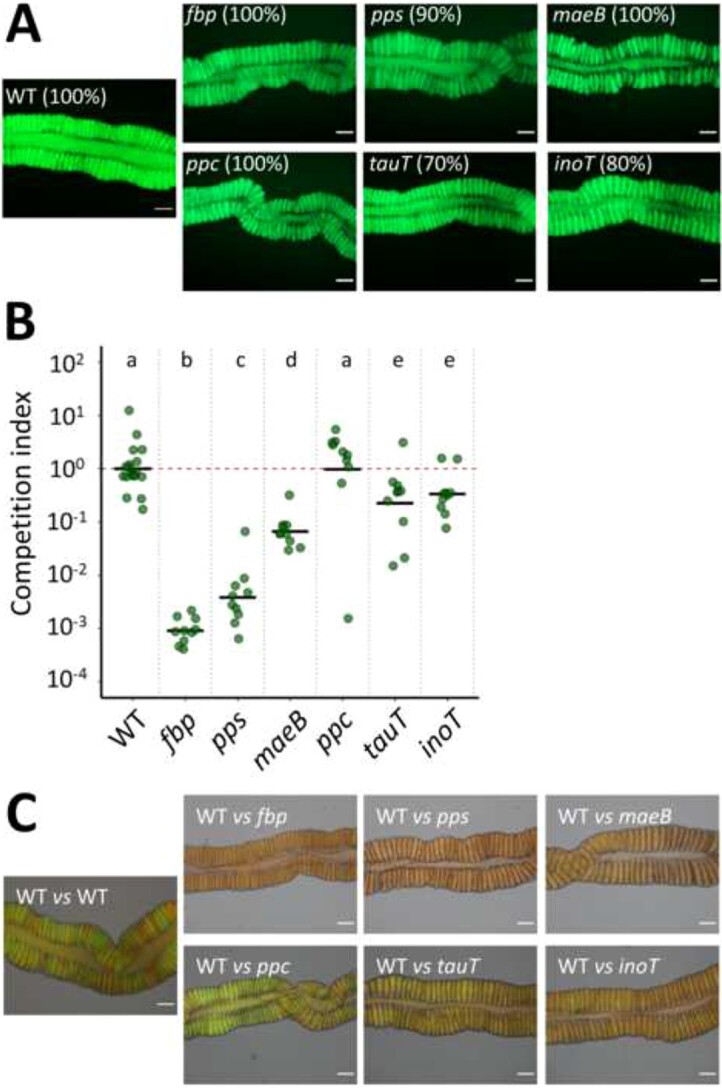
Ability of *C. insecticola* mutants to colonize the *R. pedestris* M4 midgut region; midguts were analyzed at 5 dpi; (A) colonization capacity of WT and mutant strains in single-strain infection conditions were determined by microscopy observation with an epi-fluorescence microscope; one representative image is shown for each condition; infection rate (%) indicates the proportion of infected animals with indicated strains (*n* = 10); (B) colonization capacity of strains in coinfection conditions; *Riptortus pedestris* was infected with an equal mix of mScarlett-labeled *C. insecticola* WT and the indicated GFP-labeled WT or mutant strains; relative abundance of the two strains in the M4 midgut regions at 5 dpi was determined by flow cytometry on dissected intestines; the competition index expresses for all samples the ratio of the indicated mutant to WT, corrected by the ratio of the inoculum, which was in all cases close to 1 (mutant bacteria/WT bacteria)/(inoculum mutant bacteria/inoculum WT bacteria); each dot represents the competition index in an individual and the mean per mutant is indicated by a horizontal black line (*n* = 10); CI is competition index; different letters indicate statistically significant differences (*P* < .05); statistical significance was analyzed by Kruskal–Wallis test, Dunn *post hoc* test, and Benjamini–Hochberg correction; (C) microscopy observation with an epi-fluorescence microscope of competition assays between *C. insecticola* WT (RFP) and mutants (GFP) as in panel B; one representative image is shown for each condition. Scale bars in panels A and C are 40 μm.

Next, the capacity of the mutants to colonize the gut symbiotic organ in *R. pedestris* in mono-inoculation and in co-inoculation with the WT strain was tested (see Materials and methods). The mutants were all able to colonize the insect symbiotic organ in mono-inoculation conditions (Fig. 6A). In co-inoculation, we calculated a competitive index to estimate the fitness difference between mutants and WT bacteria (Fig. 6B and C). For the three mutants with insertions in key enzymes of the gluconeogenesis, in particular the *fbp* and *pps* mutants, we observed that their capacity of colonization of the symbiotic organ was strongly affected in presence of the WT bacteria in co-inoculation experiments, implying that these three genes play a key role in the bacterial fitness for insect crypt colonization. The *tauT* and *inoT* mutants were also clearly outcompeted by the WT although to a lesser extent than the three first mutants, suggesting that taurine and inositol are available nutrients in the crypt region. In contrast, the mutant in the glycolytic enzyme *ppc* was equally capable as the WT to colonize the insect symbiotic organ, implying the absence of glycolytic carbon sources.

## Discussion

The availability of a transposon mutant library of *C. insecticola* and the genome-wide identification of essential and conditionally fitness genes constitutes a step toward the characterization of the fitness landscape of this bacterium in its different living environments, which include persistence in soil, colonization of the symbiotic organ of insects, as well as interactions with other organisms present in the natural environment of this bacterium like soil microbes and plants.

Chromosome 1 carries the large majority of genes conserved between *Burkholderia s.l.* species, whereas the other replicons are more enriched in genes with limited species distribution [50]. Accordingly, we found that nearly all the essential genes of *C. insecticola* are located on Chromosome 1. This observation is moreover in agreement with the plasmid origin of the other two chromosomes, which therefore can be considered as chromids. Probably, ancestral plasmids acquired genes, including essential ones, from Chromosome 1 after their capture by the ancestor of *C. insecticola*, giving rise to the present essential chromids. Since Chromosome 3 contains only two essential genes besides the replication/partitioning genes, suggesting that, after translocation of these two essential genes to Chromosome 1 or 2, this replicon could be entirely cured from the *C. insecticola* genome. In agreement, it was shown that Chromosome 3 in *B. cenocepacia* strains can be efficiently cured [51, 52]. The absence of essential genes in the plasmids is in line with the demonstration that Plasmid 2 can be removed from the bacterium without affecting its fitness [13] and further suggests that the large Plasmid 1 can be removed as well without affecting viability. A reduced-genome engineered strain lacking Chromosome 3, Plasmid 1, and Plasmid 2 could be a powerful platform that can be used for gene discovery.

Among the 498 essential genes of *C. insecticola*, we found only a surprisingly small portion of them conserved among related species from the *Burkholderia s.l.*. However, despite the fact that essential genes code for fundamental cellular functions, essential gene sets are known to be specific to each bacterium, and they can even vary among strains belonging to the same bacterial species, similarly as we find here for the three analyzed *B. cenocepacia* strains [53-55]. Non-orthologous gene displacement is one explanation that has been put forward for the absence of conservation of essential genes among bacteria. This concept proposes that essential pathways or genes are replaced or coexist with functional equivalents with no DNA homology and different evolutionary origin [55]. Gene duplication on the other hand could render genes nonessential because of redundancy, whereas the encoded function remains essential. Gene duplication is observed only for a few genes that are nonessential in *C. insecticola* but essential in related species, suggesting that non-orthologous gene displacement is accounting for a large part of the differences among the essential gene sets in *Burkholderia s.l.* species. Moreover, it should be mentioned that different Tn-seq techniques and bioanalysis tools were employed for the determination of the essential gene sets of these eight species. This heterogeneity in analysis methods may create biases in the identification of common essential genes and potentially their number is in reality higher than that we determined here.

We showed here that the transposon library in *C. insecticola* in combination with the Tn-seq method provides robust fitness data at the whole-genome level and that it can be used efficiently to identify conditionally essential genes in this species. We identified genes that are specifically essential for growth in media with gluconeogenic or glycolytic carbon sources, with rhamnose, mannitol, myo-inositol, and taurine and confirmed their metabolic function by mutagenesis for a subset of them. Our Tn-seq study thus contributed to annotate genes with a previously unknown role as exemplified by the chloride channel required for taurine utilization.

The phenotypic characterization of constructed mutants further revealed that the *C. insecticola* bacteria in the gut are fed by the insect with gluconeogenic carbon sources, including taurine, as well as with myo-inositol but not with glycolytic nutrients. It appeared that if the use of gluconeogenic substrates is crucial for an efficient colonization, the capacity to utilize individual compounds like taurine or myo-inositol has a less strong impact because mutants, which are unable to import these molecules, are still able to partially colonize the insect gut in competition with the WT. It should be noted that phosphoenolpyruvate carboxylase encoded by *ppc* is required for growth on myo-inositol as a sole carbon source (Fig. 4D), whereas *ppc* is not required for gut colonization. Therefore, myo-inositol in the gut is unlikely to fuel the TCA cycle via *ppc* but is rather assimilated into a different pathway. Moreover, all the tested mutants were able to colonize the crypt region in the absence of competition with WT bacteria. Together, this suggests that the insect is providing multiple but specific nutrients to the bacteria. Thus, the nutritional exchange in the symbiosis between *C. insecticola* and *R. pedestris* is complex [13]. Our study demonstrates that Tn-seq can contribute to dissect this process in detail. An exciting possibility, worth to be investigated in the future, is that the feeding of the crypt bacteria with specific nutrients tailors the symbiont metabolic fluxes toward an optimized production of particular beneficial metabolites for the host.

In conclusion, the Tn-seq approach is a powerful tool for whole-genome genetic screens in *C. insecticola* and our strategy, which consisted in Tn-seq analyses in MM with different nutrient sources proposed by transcriptomics to be important during symbiosis, is one of the few studies that have elucidated physiological contributions, which microbial symbionts receive from their host. Future Tn-seq experiments with *C. insecticola* in other *in vitro* conditions or in its natural environments, like in the insect gut, soil, rhizosphere, or cocultivation with other microorganisms, will strongly help to better understand the different lifestyles of this bacterium. Defining the genetic repertoires that determine the fitness of *C. insecticola* in these environments will highlight pre- and post-adapted traits of the *Caballeronia* symbiont to the insect’s gut environment.

## Supplementary Material

Supp_data_1_MM_media_ycad001

Supp_data_2_essential_genome_CI_ycad001

Supp_data_3_Comparison_6_Burkholderia_v2_ycad001

Supp_data_4_resampling_output_ycad001

Supplementary_information_revision_ycad001

## Data Availability

Tn-seq sequencing data were deposited in the SRA (BioProject accession n° PRJNA1004562 and PRJNA890438). All other data generated or analyzed during this study are included in this published article and its supplementary information files.
