# Supplementary material for: Transposon sequencing reveals the essential gene set and genes enabling gut symbiosis in the insect symbiont Caballeronia insecticola
Source: ISME Commun. 2024 Jan 10;4(1):ycad001. doi: 10.1093/ismeco/ycad001 (PMC10809759; doi:10.1093/ismeco/ycad001)
Supplement: Supplementary_information_revision_ycad001 [file supplementary_information_revision_ycad001.pdf]

Transposon sequencing reveals the essential gene set and genes enabling gut symbiosis in the insect symbiont *Caballeronia insecticola*

Romain Jouan, Gaëlle Lextrait, Joy Lachat, Aya Yokota, Raynald Cossard, Delphine Naquin, Tatiana Timchenko, Yoshitomo Kikuchi, Tsubasa Ohbayashi, Peter Mergaert

Supplementary information

Content:

Supplementary Figures s1 to s3

Supplementary Table and Datasets 1 to 4

Supplementary Text

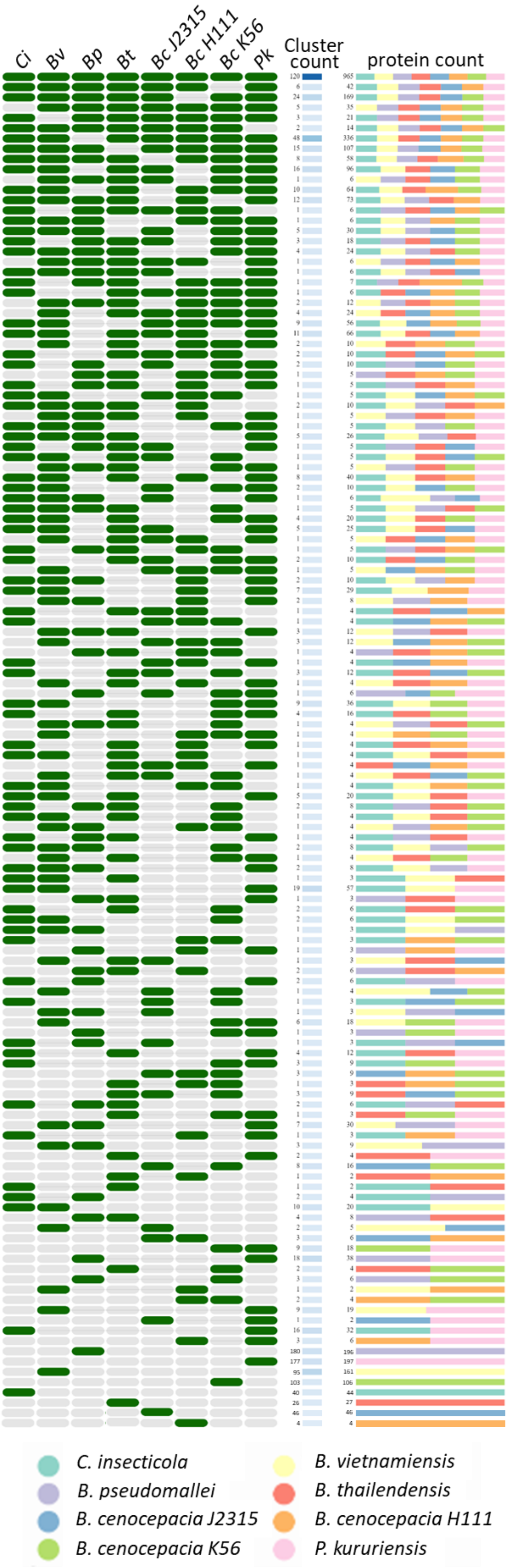

Supplementary Figure s1. Homology-based comparison of essential genes shared between eight *Burkholderia s.l.* species using OrthoVenn2. The essential gene sets of the eight indicated species are pooled and orthologous groups (clusters) are created. The presence of clusters in the species are indicated by green cells and absence with grey cells. Between species shared cluster numbers are indicated in the cluster count column and by the colour code from dark to light blue of the corresponding cell. The protein count indicates per cluster the number of proteins present in each species according to the colour key.

	C. insecticola	*B. vietnamiensis*	*B. pseudomallei*	*B. thailandensis*	*B. cenocepacia* J2315	*B. cenocepacia* H111	*B. cenocepacia* K56
B. vietnamiensis	386						
B. pseudomallei	238	248					
B. thailandensis	333	322	225				
***B. cenocepacia* J2315**	290	290	196	272			
***B. cenocepacia* H111**	288	298	195	274	247		
***B. cenocepacia* K56**	321	318	214	291	293	259	
P. kururiensis	407	412	272	338	293	301	332

Supplementary Figure s2. Pairwise comparison of essential gene sets in eight analysed *Burkholderia s.l.* species.

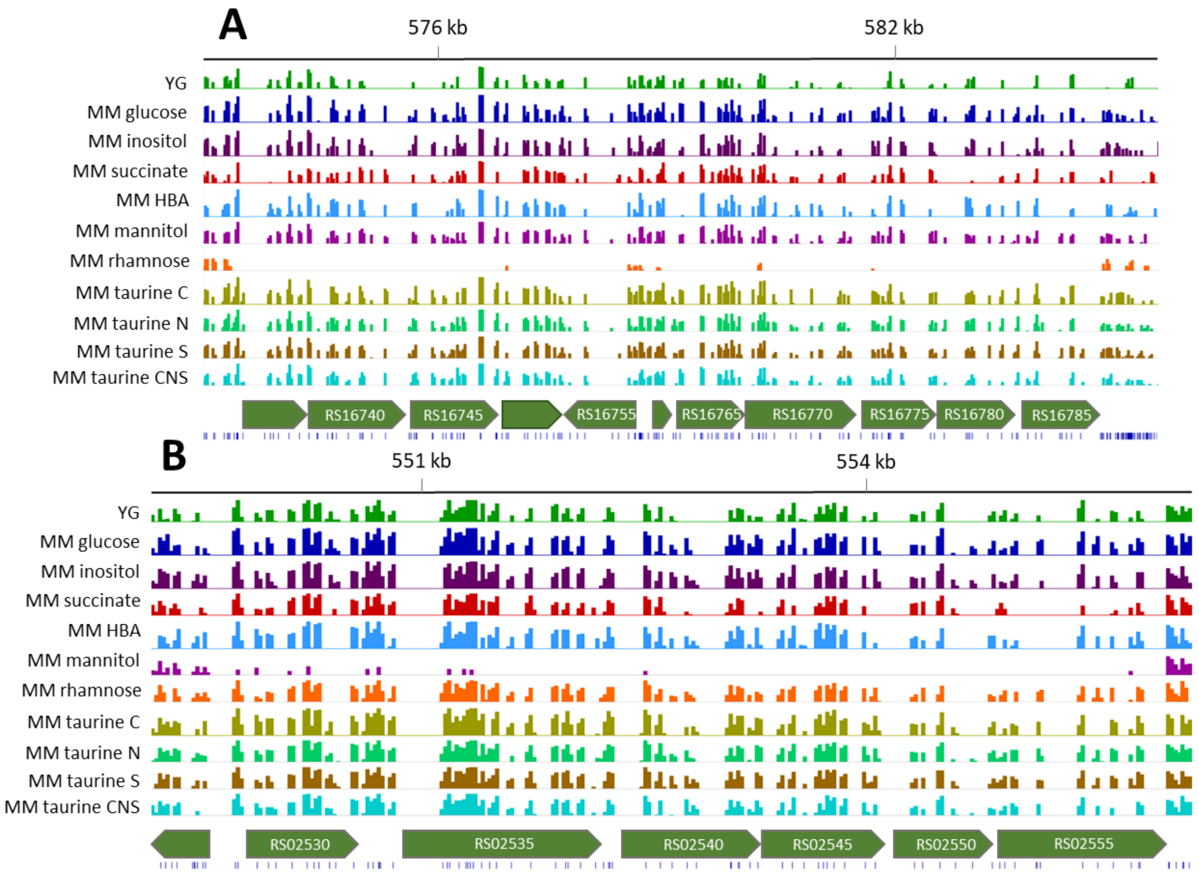

Supplementary Figure s3. IGV plots of genomic regions carrying condition-specific fitness genes. **A.** Rhamonose utilisation genes. **B.** Mannitol utilization genes. Tracks, from bottom to top: Position of TA sites (blue bars); Gene organization in the region of interest with fitness genes (in green) and their flanking neighbours (in grey); Histograms of insertion counts at TA sites for the indicated experimental conditions; Genome positions (in kb) on the chromosome 1. HBA, 3-hydroxybutyric acid; taurine C, taurine as carbon source; taurine N, taurine as nitrogen source; taurine S, taurine as sulphur source; taurine CNS, taurine as carbon, nitrogen and sulphur source.

Supplementary Table and Datasets

Supplementary Table s1. Primers used in this study.

Supplementary Dataset s1. Composition of minimal media used for Tn-seq screens and growth curves.

Supplementary Dataset s2. The essential genes of *C. insecticola* identified by Transit analysis and results of HMM analyses for the YG, MM glucose and MM succinate conditions.

Supplementary Dataset s3. Essential genes in *C. insecticola* common between the eight Burkholderia s.l. species and Orthovenn2 clusters.

Supplementary Dataset s4. Transit resampling analysis of Tn-seq data from growth on various nutrients.

Supplementary text

Generation of a *C. insecticola* RPE75 *Himar1* transposon library

The *E. coli* MFDpir strain carrying the plasmid pSAM_EC with a modified *Himar1* mariner transposon, containing the *nptII* kanamycin resistance gene [1] was used as a donor strain for transposon mutagenesis. The donor strain *E. coli* MFDpir pSAM_Ec and the recipient strain *C. insecticola* RPE75 were grown in 50 mL liquid cultures until exponential growth phase with a final OD_{600nm}≈1. Bacteria were washed twice by centrifugation of cultures at 4000 rpm for 10 minutes at 4°C and resuspension of pellets in fresh medium. Final pellets were resuspended in 1 mL fresh medium to obtain an OD_{600nm}≈50. For conjugation, the donor and the recipient strain were mixed at a 1:1 ratio, spotted per 100 µL on YG agar plates supplemented with 300 µg/mL DAP and plates were incubated at 28°C. After 1 hour of incubation, 1 ml of YG medium was added per conjugation spot to recover bacteria. Dilution series of this bacterial mix were plated on a selective medium carrying Rif (selection of RPE75) and Km (selection of the transposon) and subjected to colony forming units (cfu) counting to assess the number of independent bacterial mutants obtained by the mutagenesis, which was estimated to be about 2.5×10^8 clones. In parallel, the totality of the remaining bacterial suspension was spread on 100 YG agar plates (100 µL per plate) supplemented with Rif and Km to obtain the *C. insecticola* transposon mutant population. After 2 days of incubation at 28°C, the transposon library was resuspended from the agar plates in fresh liquid YG medium. The suspension was adjusted to 20 % glycerol, aliquoted per 1 mL and stored at -80°C. The titer of this library was about 2×10^{10} cfu/mL for a total of 127,5 mL.

Before further use, a quality control was performed on the library. The presence of the mariner transposon and the absence of the transposon donor plasmid pSAM_Ec were verified

by PCR on 20 randomly selected clones of the obtained transposon library. The transposon borders of these clones were amplified by PCR as described below and inserted into the pGEM-T Easy plasmid (Promega). For each of these 20 pGEM-T constructs, 10 clones were sequenced. This verification confirmed that each initial *C. insecticola* clone carried a single *Himar1* transposon insertion, that the two borders of the transposon were obtained and that the each of the 20 randomly selected clones carried a transposon in a distinct genomic location, distributed over the genome.

DNA extraction and preparation of the high-throughput sequencing libraries

Genomic DNA was extracted from the bacterial pellets using the MasterPure™ Complete DNA and RNA purification kit (Epicentre) according to the manufacturer's instructions. Samples were cleaned-up by removing RNA according to instructions. Samples of 10 µg DNA were digested for one hour at 37°C with 1 µL of *Mme*I enzyme (2000 U/mL, New England BioLabs), in a total volume of 250 µL mix supplemented with 25 µL of 10X CutSmart buffer (New England BioLabs) and 10 µL of S-adenosine-methionine (1.5 mM, New England BioLabs). Subsequently, 1 µL of FastAP Thermosensitive Alkaline Phosphatase (1 U/µL, ThermoScientific) was added to the digestion mixes and samples were incubated for one additional hour at 37°C. The enzymes were then heat-inactivated at 75°C for 5 minutes. Digested DNA samples were purified from the reaction mix using the QIAquick PCR purification kit (QIAGEN). 700 ng of digested DNA was ligated to specific barcoded adaptors (5 µM) (Table s1) using T4 DNA ligase (1 U/µL, ThermoScientific) in a final volume of 20 µL and incubated overnight at 16°C. The double stranded adaptors were prepared beforehand by mixing 25 µL of each corresponding single stranded primer at 200 µM (Table 1) and 1 µL of TrisHCl (100 µM, pH 8.3), denaturing the primers in the mixture at 92°C for 1 min and promoting the annealing of the complementary

primers by gradual cooling of the samples (2°C per min) in a PCR thermocycler. Transposon borders were subsequently amplified by PCR from the adapter-ligated DNA samples using 1 µL of them as template. The PCR was performed for 22 cycles using the EuroBio Taq polymerase (5 U/µL, reference GAETAQ00-4W) in a final volume of 20 µL according to the manufacturer's instructions, with 0.5 µM of the forward P7 Illumina primer and 0.5 µM of the reverse P5 Illumina primer (Table s1 for primer sequences). The amplified products (130 bp) were separated on a 2.5 % agarose gel by electrophoreses and purified from the gel using the QIAquick gel extraction kit (QIAGEN). The concentration and the quality control of these Tn-seq Illumina sequencing library samples were assessed using Qubit fluorometric quantification (ThermoFisher) and a Bioanalyzer instrument (Agilent), respectively.

Sequencing and sequence data treatment

Up to 20 Tn-seq samples were mixed in equimolar amounts and sequenced by an Illumina NextSeq 500 instrument with 2 x 75 paired-end run at the I2BC sequencing platform (CNRS Gif-surYvette, France). The generated data were demultiplexed using bcl2fastq2 software (bcl2fastq v2.15.0; Illumina, San Diego, USA) and FASTX-Toolkit (http://hannonlab.cshl.edu/fastx_toolkit). The 3' transposon sequence was trimmed using Trimmomatic [2], and reads with a length of 75 nucleotides were removed (reads without the transposon insertion). After the trimming step, reads with a length between 19 and 23 bp were reverse-complemented and only the reads starting with TA dinucleotides were mapped using Bowtie (bowtie-1.1.2) [3,4] to the reference genome of *C. insecticola* [5] (accession n° NC_021287.1 (chromosome 1), NC_021294.1 (chromosome 2), NC_021288.1 (chromosome 3), NC_021289.1 (plasmid 1), NC_021295.1 (plasmid 2)). BAM output files were sorted with Samtools (http://www.htslib.org/). FeatureCounts [6] was used to evaluate the number of

reads per gene. BAM output files were converted with Samtools on the Galaxy server (https://usegalaxy.org/) into non-binary SAM files, the appropriate format to use for further analysis.

Identification of (conditionally) essential genes by Transit software

Tn-seq sequencing data was handled by TRANSIT Version 3.2.0. It provides an easy to use graphical interface and access to several different Tn-seq data analysis methods that allow the user to determine essentiality within a single condition (Hidden Markov Model analysis) as well as between two conditions (Resampling analysis) [7].

The Hidden Markov Model (HMM) analysis from Transit software allows determining fitness of each TA site and each gene of the genome of interest in a single condition. By this analysis, genes are classified as non-essential (NE), growth defect (GD), growth advantage (GA) or essential (ES). The selected HMM parameters in TRANSIT were 10 % cutoff, TTR normalization and sum of replicates. The resampling analysis from Transit software compares the number of transposon insertions in a control condition to the number of transposon insertions in the experimental condition for each gene of the genome of interest. For each gene, a *p*-value to assess the significance of the difference with the control condition and a log2-fold-change expressing the importance of these differences is obtained. The parameters used in the resampling analysis were 10 % cutoff, 10 000 samples, pseudo-count of 5 and TTR normalization. Selected cut-off values for significant fitness change were $|\log_2\text{-fold-change}| > 1.58$ and $p < 0.05$. Each experimental condition was compared to the MM with glucose as the reference condition.

The Integrative Genomics Viewer (IGV) software, an interactive tool for the visual exploration of genomic data, was used to visualize the number of insertions per insertion sites in specific genes of interest [8].

Creation of fluorescent protein tagged *C. insecticola* strains

A mScarlett-I-tagged strain of *C. insecticola* was created by introducing a Tn7-Scarlet transposon. The Tn7-Scarlet donor strain S17-1λpir.pMRE-Tn7-135, the helper strain WM3064.pUX-BF13 and *C. insecticola* were grown overnight in LB with DAP if appropriate and YG respectively. Overnight cultures were diluted in 10 mL fresh medium using 0.2 mL of the overnight cultures and grown at 180 rpm until reaching a final $OD_{600nm}<1$. The cultures were washed twice in fresh YG medium without antibiotics by centrifugation at 4000 rpm for 10 minutes. Bacterial pellets were resuspended in fresh YG medium to obtain a final $OD_{600}\approx5$ to 10. Then the donor, helper and *C. insecticola* strains were mixed at a 1:1:1 ratio. 150 µL of the conjugation mix was spotted on YG agar plates with DAP and 0.1 % of L-arabinose to induce transposition. After overnight incubation at 28°C the spots on the plates were resuspended in 1 mL of YG medium. Ten-fold dilution series were made and 50 µL of each dilution were plated out on YG plates complemented with Cm. DAP was not added to counterselect the *E. coli* strains from the mixture. Selected colonies were screened for mScarlet-I expression by UV light illumination and they were purified again on YG complemented with Rif and Cm.

Insect rearing and inoculation tests

The bean bug, *R. pedestris,* was originally collected from Japan, from soybean field in Tsukuba, in 2007. The insects are maintained in the laboratory and are reared in plastic boxes at 25°C under a long-day regimen (16 h light, 8 h dark) and fed with soybean seeds and distilled water

containing 0.05 % ascorbic acid (DWA). After hatching, insect eggs were transferred into sterile Petri dishes. After two days, at the second larval stage, water was removed to make insects thirsty, which facilitates the subsequent ingestion of administered bacteria. After overnight starvation, a bacterial suspension of the tested *C. insecticola* strain adjusted at 10^7 cfu/mL in sterile distilled water was provided for infection of the second instar nymphs. For co-inoculation experiments, two bacterial strains were mixed together at a 1:1, each adjusted before to 10^7 cfu/mL.

At three and five days post inoculation, insects, at the stage of the end of the second instar nymphs or the third instar, respectively, were dissected. Dissections were performed in sterile PBS (137 mM NaCl, 8.1 mM Na_2HPO_4, 2.7 mM KCl, and 1.5 mM KH_2PO_4, pH 7.5) containing 0.01 % of Tween 20 under a Stemi 508 binocular microscope equipped with an Axiocam 208 color camera (Zeiss). The M4 region of the midgut was collected using fine forceps and assembled on glass slides for microscopy observations (Nikon Eclipse 80i). The colonization rate of the inoculated insects was estimated by fluorescent signal detection of colonizing bacteria with GFP or mScarlett-labelled fluorescent proteins. Merged fluorescence pictures were obtained with GIMP version 2.10.32. Then M4 region samples were homogenized in PBS solution and bacteria in suspension were counted by flow cytometry, using the fluorescent tags to determine the relative abundance of the two inoculated strains. Flow cytometry was performed on a CytoFlex S instrument operated by CytExpert 2.4.0.28 software (Beckman Coulter). Gating by the forward-scatter (FSC) and side scatter (SSC) dot plot permitted to collect signals specifically derived from bacteria. Doublets were discarded using the SSC_Area-SSC_Height dot plot. GFP fluorescence was excited by a 488-nm laser and collected through a 525/40 nm band pass filter; RFP fluorescence was excited by a 561-nm laser and collected

through a 610/20 nm band pass filter. Data acquisition for a total of 50 000-100 000 bacteria was performed for each sample. Thresholds for considering positive events for GFP and RFP were determined using non-fluorescent control bacteria. Data was treated by CytExpert and Excel software. Statistical analysis were performed in R using a Kruskal wallis test, with pairwise Wilcoxon test as post hoc test and the Benjamini-Hochberg p.adjust method ($p < 0.05$).

References

1. Wiles TJ, Norton JP, Russell CW, Dalley BK, Fischer KF, Mulvey MA. Combining quantitative genetic footprinting and trait enrichment analysis to identify fitness determinants of a bacterial pathogen. PLoS Genet. 2013;9(8):e1003716. doi: 10.1371/journal.pgen.1003716. Epub 2013 Aug 22. PMID: 23990803; PMCID: PMC3749937.
2. Bolger AM, Lohse M, Usadel B. Trimmomatic: a flexible trimmer for Illumina sequence data. Bioinformatics. 2014 Aug 1;30(15):2114-20. doi: 10.1093/bioinformatics/btu170. Epub 2014 Apr 1. PMID: 24695404; PMCID: PMC4103590.
3. Langmead B, Trapnell C, Pop M, Salzberg SL. Ultrafast and memory-efficient alignment of short DNA sequences to the human genome. Genome Biol. 2009;10(3):R25. doi: 10.1186/gb-2009-10-3-r25. Epub 2009 Mar 4. PMID: 19261174; PMCID: PMC2690996.
4. Li H, Durbin R. Fast and accurate long-read alignment with Burrows-Wheeler transform. Bioinformatics. 2010 Mar 1;26(5):589-95. doi: 10.1093/bioinformatics/btp698. Epub 2010 Jan 15. PMID: 20080505; PMCID: PMC2828108.
5. Shibata TF, Maeda T, Nikoh N, Yamaguchi K, Oshima K, Hattori M et al. Complete Genome Sequence of Burkholderia sp. Strain RPE64, Bacterial Symbiont of the Bean Bug Riptortus

pedestris. Genome Announc. 2013 Jul 5;1(4):e00441-13. doi: 10.1128/genomeA.00441-13. PMID: 23833137; PMCID: PMC3703598.

6. Liao Y, Smyth GK, Shi W. featureCounts: an efficient general purpose program for assigning sequence reads to genomic features. Bioinformatics. 2014 Apr 1;30(7):923-30. doi: 10.1093/bioinformatics/btt656. Epub 2013 Nov 13. PMID: 24227677.

7. DeJesus MA, Ambadipudi C, Baker R, Sassetti C, Ioerger TR. TRANSIT--A Software Tool for Himar1 TnSeq Analysis. PLoS Comput Biol. 2015 Oct 8;11(10):e1004401. doi: 10.1371/journal.pcbi.1004401. PMID: 26447887; PMCID: PMC4598096.

8. Robinson JT, Thorvaldsdóttir H, Winckler W, Guttman M, Lander ES, Getz G, Mesirov JP. Integrative genomics viewer. Nat Biotechnol. 2011 Jan;29(1):24-6. doi: 10.1038/nbt.1754. PMID: 21221095; PMCID: PMC3346182.
